# MicroRNAs in Pancreatic Cancer: biomarkers, prognostic, and therapeutic modulators

**DOI:** 10.1186/s12885-019-6284-y

**Published:** 2019-11-21

**Authors:** Afra Z. Daoud, Eoghan J. Mulholland, Grace Cole, Helen O. McCarthy

**Affiliations:** 1 School of Pharmacy, Queen’s University Belfast, 97 Lisburn Road, Northern Ireland, BT9 7BL UK; 2 Gastrointestinal Stem Cell Biology Laboratory, Wellcome Trust Centre for Human Genetics, University of Oxford, Oxford, OX3 7BN UK; 3 Genome Sciences Centre, British Columbia Cancer Agency, Vancouver, British Columbia V5Z 1L3 Canada; 4 Department of Pathology and Laboratory Medicine, University of British Columbia, Vancouver, V6T 2B5 Canada

**Keywords:** Pancreatic Cancer, microRNA, Biomarker, Prognosis, Chemoresistance, Gene therapy

## Abstract

**Abstract:**

A severe lack of early diagnosis coupled with resistance to most available therapeutic options renders pancreatic cancer as a major clinical concern. The limited efficacy of current treatments necessitates the development of novel therapeutic strategies that are based on an understanding of the molecular mechanisms involved in pancreatic cancer progression. MicroRNAs (miRNAs) are non-coding small RNAs that regulate the expression of multiple proteins in the post-translation process and thus have promise as biomarkers, prognostic agents, and as advanced pancreatic therapies.

Profiling of deregulated miRNAs in pancreatic cancer can correlate to diagnosis, indicate optimal treatment and predict response to therapy. Furthermore, understanding the main effector genes in pancreatic cancer along with downstream pathways can identify possible miRNAs as therapeutic candidates. Additionally, obstacles to the translation of miRNAs into the clinic are also considered.

Distinct miRNA expression profiles can correlate to stages of malignant pancreatic disease, and hold potential as biomarkers, prognostic markers and clinical targets. However, a limited understanding and validation of the specific role of such miRNAs stunts clinical application. Target prediction using algorithms provides a wide range of possible targets, but these miRNAs still require validation through pre-clinical studies to determine the knock-on genetic effects.

**Graphical abstract:**

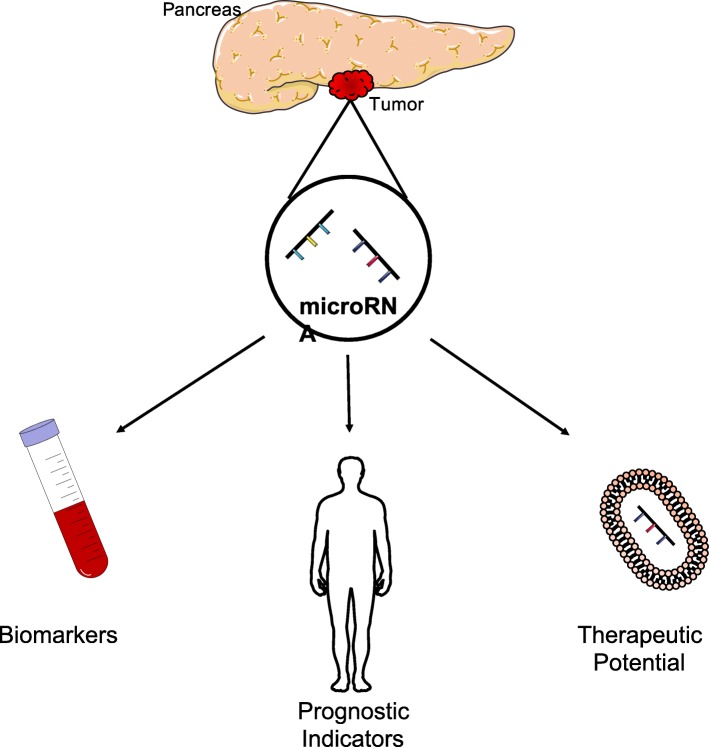

## Background

### Introduction to Pancreatic Cancer

Cancer is a devastating disease, and of the 18 million diagnosed globally in 2018, approximately 500,000 cases were pancreatic [1]. Furthermore, pancreatic cancer (PC), in all forms, has the lowest survival rate of any cancer type; with less than 1% overall 10-year survival, and 3% overall 5-year survival based on statistics of patients from England and Wales [2]. Although significant progress has been made in the development of novel cancer therapies, PC survival rates have failed to improve in the last 40 years [2]. A fundamental reason for this is that PC typically presents as an advanced disease with extensive metastatic deposits that arise in the liver [3]. Additionally, surgeons are often reluctant to conduct resections on local tumours due to growth around vital arteries and para-aortal metastases [4, 5].

The evolution of genetic mutations required for metastasis can take over a decade to come into fruition from the initial primary non-metastatic cell within the pancreas [3]. This means that if the disease is detected within the first couple of years of tumorigenesis, there is a significantly improved chance of disease control with effective treatment [6]. Abdominal pain and abnormal digestive patterns have been reported to be recurring in 70% patients with PC diagnosis, which in later stages become more apparent with specific patterns identified [7].

Diabetes mellitus (DM) is unsurprisingly found in two thirds of PC patients. Studies consistently show an increase in the risk of PC development with type 2 DM [8]. Furthermore, in a recent study by Wang et al. [9], DM was linked with increased metastasis in PC patients. These findings were linked to an increased inflammatory response accompanied by high glucose levels promoting PC progression [9]. Nonetheless, the pancreatic tumour itself can be diabetogenic resulting in dysfunctional β-cells, required for insulin production [10]. This explains the association of PC with 10% of new-onset diabetes [11], as well as highlighting newly developed DM as a possible PC symptom.

Taken together, these facts indicate that PC is still a challenge in terms of diagnosis, treatment and prognostic outlook. This review therefore will explore the molecular changes which can occur in PC development and progression, and how (microRNA) miRNA can play a role in terms of, disease screening, prognoses and new therapeutic options.

## Main text

### Molecular changes during Pancreatic carcinogenesis

The development of PC is a multistage process that involves alterations in well-characterised genes [12]. One of the most commonly mutated oncogenes is the Kirsten Rat Sarcoma (K-RAS) gene, detected in 90% of PC cases [11].

**Fig. 1:**
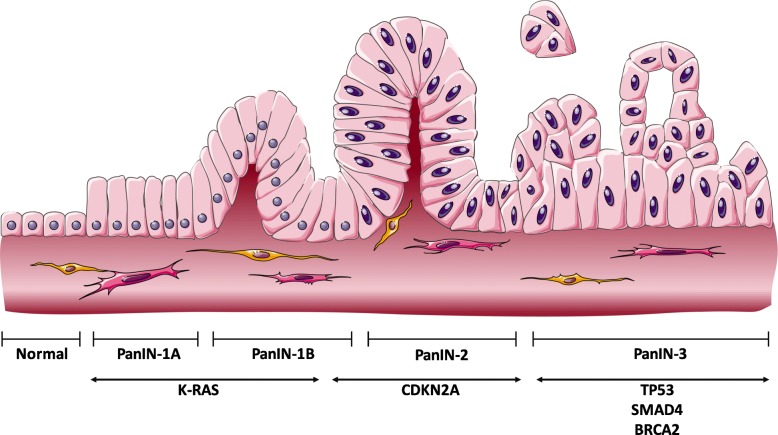
Schematic representation of the development of PC and associated genes in each stage. The precursor lesions (PanINs) give rise to PC in a multistage process that is mediated through consecutive genetic mutations starting with early K-RAS oncogenic activation and ending in multiple tumour suppressors silencing. Source: MS Powerpoint

Typical PC tumour suppressor mutations can be found in cyclin-dependent kinase (CDKN2A), tumour protein 53 (TP53), mothers against decapentaplegic homolog 3 (SMAD3), and breast cancer A2 (BRCA2) [13]. These genetic mutations result in histological and morphological abnormalities within the ductal cells of the pancreas, forming papillary-like structures. These papillary lesions then transform into more complicated preneoplastic lesions known as pancreatic intraepithelial neoplasia (PanIN) [14]. The formation and development of PanINs occur in a stepwise linear manner that is concurrent with the aforementioned genetic mutations. As a result, more complicated structures develop, progressing to the invasive (carcinoma) tumour cells (Fig. 1). Thus, PanINs are classified based upon cellular morphology, as PanIN-1A, −1B, − 2 or − 3. PanIN-1A and PanIN-1B are more elongated compared to the normal ductal cells and cells with flat structures fall under the PanIN-1A subgroup. Both A and B subgroups are linked by the early mutation of the K-RAS oncogene. With the inactivation mutation of CDKN2A, the lesions progress and acquire moderate nuclear abnormality, forming PanIN-2. Ultimately, nuclear atypia ensues with the budding of cells into the ductal-like structure in the late lesions known as PanIN-3. These late lesions arise as a result of inhibitory mutations of p-16 and SMAD-4 and are also dubbed as in situ carcinoma [15]. PanINs can also be divided into low-grade or high-grade, with a high rate of occurrence of low-grade in the early stages of PC and excessive tumour invasion happening in the later stages [16].

### Oncogene activation

K-RAS is a member of the Ras family, a guanosine triphosphate (GTP) family of bound proteins. Active Ras proteins bind to GTP and are responsible for proliferation, cell survival and cytoskeletal remodelling through the regulation of several downstream modulators. The activity of Ras is then terminated through the hydrolysis of GTP into guanosine diphosphate (GDP), mediated by GTPase activating protein (GAP) [17].

Mutations arise in the K-RAS oncogene in glycine 12 (G12), glycine (G13), or glutamine (Q61L) resulting in sustained K-RAS activity through maintaining the GTP-bound active form of the protein [18]. This results in persistent activation of the downstream signalling pathways, which will be translated in a typical cancerous cell phenotype including aberrant proliferation, microenvironment alterations, apoptosis suppression and cell survival.

To study the impact of mutated K-RAS on tumour progression, Collins et al. [19] engineered two mouse models to have reversibly inducible K-RAS mutation that is tissue-specific. The two models are iK-Ras* and iK-Ras-p53^+/−^, in which the latter has an extra inactivation mutation of one allele of the p53 gene. These mouse models are believed to develop pancreatic lesions in a manner mimicking human disease, with PanINs developed within three weeks of K-RAS mutation induction in the iK-Ras* model. This model was used to demonstrate the key role of K-RAS in the initiation of the tumours. On the other hand, inactivation of K-RAS mutation in the iK-Ras-p53^+/−^ model resulted in tumour regression. The same response was noted by other authors using the iK-Ras mouse model [20]. They have recorded rapid tumour and stromal deterioration upon turning the K-RAS mutation off, with 50% reduction in tumour mass after 48–72 h of K-RAS genetic inhibition [20]. These findings elucidate the importance of K-RAS in maintaining carcinogenesis irrespective of any additional mutations, in a phenomenon known as “*K-RAS addiction*” [21]. Oncogene addiction materialises when a cancer requires the sustained activation of a specific oncogene, even after the incorporation of additional genetic mutations through advanced stages [22]. This phenomenon doesn’t repudiate the importance of additional mutations on tumour suppressor genes in the progression of tumours, as the tumours formed in the absence of other tumour suppressor mutations were stable with infrequent invasiveness [20, 23]. This does indicate that the K-RAS gene could be a potent therapeutic intervention in pancreatic cancer.

The two main downstream pathways of activated K-RAS include mitogen-activated protein kinase (MAPK) [24] and phosphoinositide-3-kinase (PI3K) [25], which have significant roles in promoting cell survival and proliferation. MAPK is a superfamily of protein kinases, including subfamilies that are identified and characterised in mammalian cells: extracellular signal-regulated kinases (ERK1/ERK2), the c-Jun N-H2 terminal kinases/stress-activated protein kinase (JNK/SAPK), and the P38 enzymes. Activation of the MAPK pathways is associated with poor prognosis in PC patients through the activation of several downstream pathways. Of these pathways, ERK1/2 [26] and P38 [27] are critical downstream modulators; however, the role of the P38 pathway is contradictory. As shown by Handa-Luca et al. who studied 99 surgically resected PC, increased cytoplasmic levels of P38 and ERK1/2 were linked with high recurrence after surgical resection of PC, and lower overall survival rates of 7 months compared to 35 months for patients showing low cytoplasmic levels [28]. Interestingly, through the analysis of 36 rapid biopsies from patients, increased levels of P38 were linked with good prognosis. This was evident as the postoperative survival was noted, with a median survival rate of 27.9 months in patients showing high levels of P38 compared to 14.7 months in those with low P38 expression [29]. The samples from this study were taken from patients presenting with all types of PC, whereas the study conducted by Handa-Luca et al. contained only pancreatic ductal adenocarcinomas patient samples. This highlights the diversity in PC and how molecules can have multiple roles.

The progressive role of inflammatory stress accompanied with the tumour microenvironment is believed to be activated through the P38 pathway [30]. Huang et al. recorded increased proliferation and invasion of a PC cell line, PANC-1, upon targeting the β-adrenergic receptors with the stress hormone, norepinephrine (NE), which was accompanied by elevated levels of the active phosphorylated P38 [30]. Yet, Ding et al. demonstrated that upon the inhibition of P38 MAPK pathway, ERK1/2 phosphorylation increased, which translated to the enhancement of PANC-1 proliferation [31].

These findings indicate that the P38-MAPK pathway has a controversial role in the development of pancreatic cancer. This contradiction may be explained by the availability of four different P38 paralogues: P38α, P38β, P38γ and P38δ (reviewed in [32]) which should be further interrogated.

### Tumour suppressor gene mutations

The CDKN2A gene encoding p16 is a tumour suppressor commonly inactivated in pancreatic cancer (90% of the cases [33]). The CDKN2A gene encodes two families of tumour suppressor proteins. Out of these families, inhibitors of the CDK4 (INK4) family is denoted mainly by p16 and p19 which are located on the same locus. However, these proteins are not considered to be isoforms of each other since they are being translated from two separate messenger RNAs (mRNA) produced by CDKN2A [34]. The most abundant inactivation mutation is believed to happen uniquely on p16. Inactivation of p16 can occur via homozygous deletion, intragenic mutations or epigenetic silencing [33, 34], which results in cell cycle disruption through the G1/S checkpoint inhibition. This disruption will result in uncontrolled transcriptional activation which will contribute to a positive feedback loop, leading to increased cell division and proliferation [35].

Another mutated tumour suppressor gene is TP53 encoding for p53, a transcription factor that is activated by cellular stress [36]. DNA damage, radiation, aberrant growth signals and some chemotherapeutic agents will cause cellular stress, thus activating the p53 pathway [37]. P53 activation results in cell division, inhibition, or in the case of extremely damaged cells, death through apoptosis [36]. The p53 mutation was recorded in 70% of PC, which mainly presents as a loss of function [38]. However, these mutations can also provide oncogenic function, as p53 silencing has been shown to increase the expression of platelet-derived growth factor receptor b (PDGFRb) which correlates directly with invasiveness and metastasis [39, 40].

Both tumour suppressor proteins p16 and p53 bind and inactivate CDK2 and 4 which impacts the cell cycle by preventing transition from G1 to S phase [41, 42]. Any inactivation of these proteins will result in cell cycle progression despite the G1/S checkpoint status ensuring continuous proliferation [43].

Another tumour suppressor gene that is commonly deregulated in PC is the pancreatic carcinoma 4 gene (DPC4, also known as SMAD4), which is upregulated in 55% of PC [41]. Loss of SMAD4 facilitates a selective growth advantage, through the regulation of the transforming growth factor-β (TGF-β) signalling pathway [42]. Upon activation of the TGF-β pathway, an intracellular cascade takes place through activation of the cell surface serine-threonine kinase receptor type II (TβRII) which will cause activation of the type I receptor through phosphorylation of the glycine- and serine-rich sequence (GS) domain located on the N-terminal of the type I receptor [42]. Receptor-activated SMADs, called R-SMADs, form trimeric complexes composed of two R-SMADs and an essential co-SMAD which is the SMAD4. These tri-complexes, translocate to the nucleus and target DNA to regulate specific genes that have anti-mitogenic and pro-apoptotic effects [41, 43]. The importance of SMAD4 in all TGF-β pathways as an essential co-factor explains the cardinal effect of SMAD4 mutations on modulating most genetic responses to the TGF-β superfamily.

Ultimately, cellular anaplasia and relapses, lymphatic invasion, postoperative recurrence, tumour size and metastasis to lymph nodes status have all been linked to mutations in p53, p16, and SMAD4 [44]. Thus, all of these genetic mutations offer great potential for gene therapy interventions. The utilisation of miRNA for the targeting of these mutations presents an opportunity to regulate multiple pathways with a single therapeutic [45].

### miRNAs

**Fig. 2:**
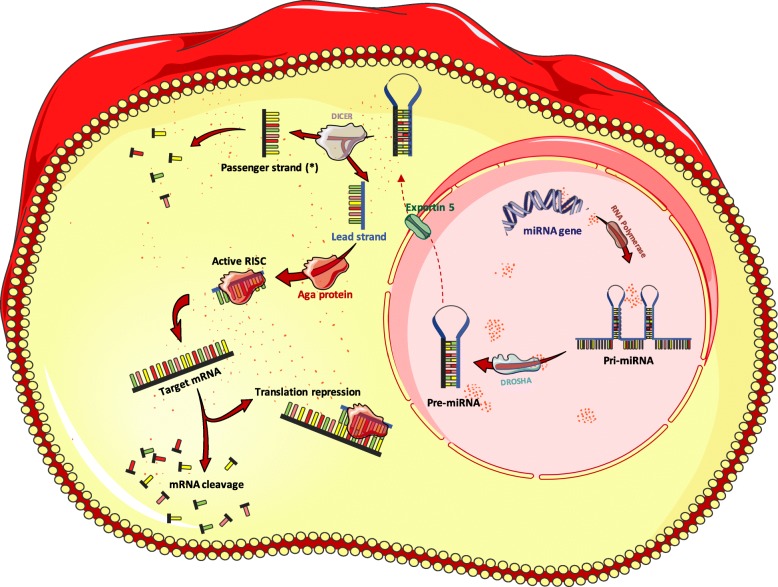
The process of miRNA biogenesis and role in post-transcriptional suppression. The biogenesis of miRNAs commences in the nucleus where the RNA polymerase II transcribes the genetic sequence encoding the miRNA to produce a primary miRNA hairpin (pri-miRNA) which is capped, polyadenylated and has a stem-loop structure. Further processing by the ribonuclease DROSHA enzyme occurs in the nucleus before the resultant 70 to 100 nt long pre-miRNA hairpin is transported to the cytoplasm via the Exportin5 protein (XPO5). Once the double stranded pre-miRNA is in the cytoplasm, RNAse DICER cleaves the molecule into two single strands, with a leading functional strand, and a passenger strand -often referred to as (*)- which will be degraded. The Ago proteins bind to the leading single stranded miRNA to form the RISC. The RISC is considered to be the functional unit in this process which facilitates the binding of miRNA into the targeted mRNA resulting in either translation repression or target degradation. Source: MS Powerpoint

MicroRNAs form a subfamily of non-coding RNAs which regulate gene expression via mRNA degradation or translatory inhibition [46]. Approximately 50% of miRNAs are encoded on non-protein-coding regions and are independently transcribed. The remaining miRNAs are encoded on introns of protein-coding transcripts. In this case, miRNAs are referred to as intergenic miRNA and will be transcribed with host genes and processed separately to produce the mature independent miRNA [47]. The biogenesis of miRNAs is summarised in Fig. 2.

Activated miRNA is incorporated within the RNA-induced silencing complex (RISC), and this multipart will bind to the target mRNA, typically on a conserved site called the seed region. This region is included within the 3′ untranslated region (3’UTR) between nucleotide 2–8, and functions as a target recognition site as the binding between the two strands has 100% complementarity [48]. Conserved seed matches are linked with more knockout potency as a single nucleotide change alters the possible targets by more than 50% [49].

The cornerstone of miRNA binding is based on 3 rules: perfect or near-perfect complementary binding in the seed region, central region mismatches or “bulges” and the reasonable complementarity throughout the full sequence of the miRNA with the target. It is important to mention that complementarity through the two sequences is particularly significant in the case of suboptimal binding in the seed region. While the full sequence of the miRNA has possible base mismatches, these mismatches do not affect the functionality of the complex. In fact, it gives the miRNA more extensive mRNA target possibilities [48]. The importance of complementary binding in the seed region is explained by the fact that it is the cornerstone in target recognition. Furthermore, the bulges facilitate the binding and functionality of the Ago protein, an essential component of the RISC required for the assembly and guidance to target mRNAs [50]. Lastly, the manner of complementary binding throughout the miRNA and the target mRNA sequence will determine the inhibitory mechanism of the translation process. If the binding occurs in a perfect complementary fashion throughout the whole sequence, complete degradation of the target mRNA will take place [51]. On the other hand, if few base pair mismatches were present during the binding process, the translation blockade will ensue through destabilisation of the target mRNA by non-splicing mechanisms. These mechanisms include decapping or deadenylation which represents the most abundant mechanism of silencing [48, 52].

Exosomes are extracellular vesicles which are endosomal in basis and typically range in size between 40 and 100 nm and function to secrete and carry biological materials between cells [53]. MiRNA can also be secreted from cells within exosomes, and these ‘exomiRNAs’ have exhibited extensive evidence of influencing cancer initiation and development. For example, exosomal miRNA-301a was found to trigger the M2 polarisation of macrophages when derived from hypoxic pancreatic cancer cells which was attributed to the activation of the PTEN/PI3Kγ pathway [54]. Interestingly, studies have shown that exomiRNAs have the potential to be novel biomarkers collected from blood serum. Studies conducted by Pang et al. showed that pancreatic cancer cells produce and secrete miRNA-155 within exosomes, with the function of activating fibroblasts [55]. Furthermore, exosomes are non-immunogenic and protect biological cargo thus make for excellent gene therapy systems. However, major hurdles of getting exosomes into the clinic include the efficient loading of miRNA into exosomes, with influencing factors including solubility and charge. Furthermore, the pathways on which any therapeutic miRNA functions would need to be extensively defined, to avoid undesirable off-target effects [56].

### miRNA in Pancreatic Cancer

miRNAs play a major role in carcinogenesis, falling into two categories: tumour suppressor miRNAs, and oncogenic miRNAs (also termed oncomiRs) [57]. The classification is dependent on the role of the target mRNAs in the tumour initiation process. Normally, oncogenes and tumour suppressor genes are regulated at an optimal activation/inhibition equilibrium. If downregulation of a specific miRNA increases the activity of a corresponding oncogene, this is identified as a tumour suppressor miRNA. On the other hand, if upregulation of an oncomiR takes place, it will result in a continuous inhibition of the target tumour suppressor gene. This uncontrolled inhibition will result in the loss of controlling specific tumour formation pathways. Deregulation in any of the miRNA types will contribute to the development of tumours [58].

Patterns of miRNA expression vary markedly between cancer types; thus, the use of miRNA expression profiles could be used as a promising non-invasive diagnostic markers. Furthermore, miRNA profiling should have superiority over using mRNA profiles as they can represent many more reliable targets [59]. Identification of a small number of miRNA has been shown to be more reliable than the data from 16,000 mRNAs with a more robust hierarchical clustering [59]. In pancreatic cancer, different patterns have been found in miRNA expression profiles, which have contributed to the development of a miRNANome between the normal and cancerous pancreas [60]. Determination of these miRNA expression profiles has been made possible through different gene profiling methods, mainly microarrays, RNA-sequencing, and RT-PCR analysis of specimens [61]. Due to the stability of miRNA in circulation, blood screening could be employed as an approach to detect specific miRNA which have been linked with stage, survival rate or aggressiveness of the disease [62–64].

Variations in the results observed among different studies are generally thought to be caused by ethnicity and regional differences [65]. Another important factor is the sampling procedure, as fine needle aspiration (FNA) results in enriched samples with a specific tumour component, disregarding other cell types during the microdissection process. This observation was illustrated by the molecular differences obtained from bulk samples compared to fine needle aspirations[66]. Comparing expression patterns among different samples could also allude to the abundance of miRNAs among different cell types that are naturally occurring in the pancreas, as well as the tumour microenvironment. For example, miRNA-375 was suggested to be linked with the islet cells [67], as the expression was high in normal pancreatic tissues compared to the cancerous and inflammatory tissues with a complete absence in representative cell lines [60].

miRNA samples can also be obtained via non-invasive routes from, peripheral blood, saliva, urine or faeces [68]. Abue et al. conducted a study to analyse the potential of miRNA-483-3p and miRNA-21 as biomarkers of pancreatic ductal adenocarcinoma from blood plasma. These plasma samples were obtained from 32 patients presenting with pancreatic ductal adenocarcinoma, 12 patients with intraductal papillary mucinous neoplasm, and 30 healthy control individuals. The levels of these miRNA were evaluated using qRT-PCR, compared between groups and the expression of each was linked clinically. The plasma expression of both miRNA-483-3p and miRNA-21 was found to be significantly higher in pancreatic ductal adenocarcinoma compared to healthy controls (*p* < 0.01). The plasma expression of miRNA-483-3p was significantly higher compared to intraductal papillary mucinous neoplasm (*p* < 0.05), and the expression of miRNA-21 was linked to advanced stage disease (*p* < 0.05) with metastases in the lymph nodes and liver (*p* < 0.01). Indeed, miRNA expression correlated with an overall lessened survival in those patients with pancreatic ductal adenocarcinoma (*p* < 0.01) [69].

### miRNAs as biomarkers

Identification of early biomarkers is essential in cases where PC surgical resection is the only curative treatment. Surgery is only feasible in 15–20% of patients who have been diagnosed with early stages of PC [70, 11]. However, the postoperative complications associated with this surgery are frequent and cases such as chronic pancreatitis or pancreatic tuberculosis are usually hard to differentiate from cancer cases [71]. To date, the serum carbohydrate antigen 19–9 (CA 19–9) has been employed as a marker for assessing clinical treatment efficacy in pancreatic cancer [72]. Limitations associated with CA 19–9 include ineffectiveness, low sensitivity and low specificity, yet it is still the only FDA approved marker in PC. Other antigens such as CEA, and CA125 were completely ineffective as early markers, but some oncologists still use them as markers of therapy responsiveness [73]. Therefore, the PC diagnostic biomarker need could be met by utilising identified miRNAs as an early screening test [74]. Advantages of using miRNAs include stability in serum, ease of non-invasive detection in circulation and a convenient screening method [75].

Lee et al. used pancreatic cancer, paired benign pancreatic tissue, normal pancreas, and pancreatitis tissues along with nine cell lines to compare miRNA expression profiles [90]. This was achieved through employment of real-time PCR profiling of > 200 miRNA precursors. The diversity of sample types used gives the study a broader spectrum of comparison and a chance of detecting the premalignant changes that occur through the conversion step from benign abnormalities to malignant tumours. Among the top aberrantly expressed miRNAs (Fold change, *P*-value) in cancer samples, miRNA-424 (56.3, 3.62E-08), miRNA-100 (36.9, 4.40E-06), miRNA-301 (34.2, 1.11E-05), miRNA-212 (22.2, 2.00E-04) and miRNA-125b-1 (23.2, 1.00E-04) were overexpressed, whereas miRNA- 345 (− 14.5, 1.44E-15), miRNA-142-P (15.4, 3.63E-07), and miRNA-139 (− 7.91, 6.79E-11) were all downregulated, relative to normal pancreatic samples. Additionally, miRNA-221, miRNA-376a, and miRNA-301 were found to be localised within the tumour cells rather than other cells in the stroma [76].

MiRNA-155 and miRNA-21 were also found to have elevated expression in the precursor lesions, linking them with histological progression features, with a specificity of miRNA-155 as a biomarker in pancreatic juices [77]. The top five upregulated miRNA included miRNA-21, miRNA-196a, miRNA-27a, miRNA-146a, and miRNA-200a as listed by Hong and Park [78] following fine needle aspiration (FNA). In comparison, the most downregulated miRNAs included miRNA-96, miRNA-217, miRNA-141, miRNA-20a, and miRNA-29c [78]. Other upregulated candidates were demonstrated via qRT-PCR by Zhang et al., and include miRNA-196a, miRNA-190, miRNA-186, miRNA-221, miRNA-222, miRNA-200b, miRNA-15b, and miRNA-95, [79]. Further evidence also showed that miRNA-21, miRNA-26b, miRNA-194, miRNA-200b, miRNA-200c, miRNA-320, miRNA-374 and miRNA-429 were upregulated in PC cell lines compared to normal pancreas cell lines [80].

**Fig. 3:**
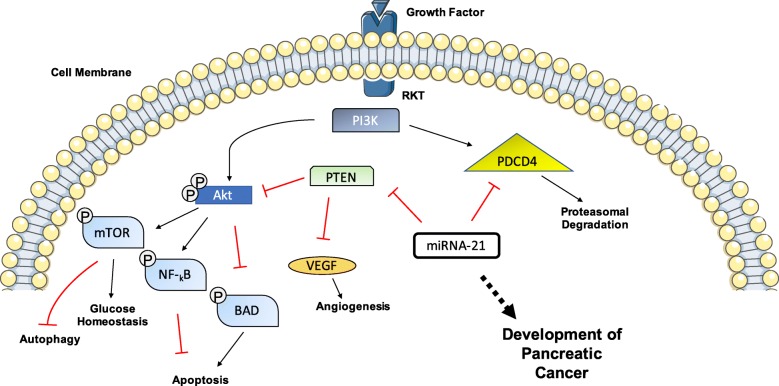
Schematic representation of mechanism of miR21 in PC development. miRNA-21 has previously been shown to target the expression of PTEN and PDCD4. Through inhibition of PTEN via miRNA-21, cell survival pathways are activated. With Akt pathway functionality heightened, the inhibition of BAD increases, which is a pro-apoptotic pathway, thus leading to a reduction in apoptosis. PTEN also inhibits angiogenesis within the VEGF pathway, thus miRNA-21 can enhance the establishment of new vasculature. Source: MS Powerpoint

Early stage K-RAS mutations, observed in PanIN lesions, can directly affect the levels of specific miRNAs as shown by the Cordelier group [67]. Upregulation of miRNA-205, miRNA-200, and miRNA-21 was detected in early adenocarcinoma lesions, through use of a KRAS(G12D) mouse model where miRNA production could be measured in pathological and nonpathologic ducts. The level of increased miRNA-21 expression was also proportional to the degree of morphological changes within the lesions [81], as quantified using qRT-PCR of PanIN samples using U6 as a housekeeper gene. The mechanism of action of miRNA-21 is summarised in Fig. 3. Upregulated miRNA-372, miRNA-146a, miRNA-204, miRNA-10a, and miRNA-10b were also detected in PC cell lines (CAPAN-1 and CFPAC1) compared to human normal pancreatic ductal epithelial cells, with more than 10-fold changes in the latter levels [82] observed using qRT-PCR.

A further study [60] showed distinct expression levels in normal pancreatic tissues. Candidate miRNAs: miRNA-141, miRNA-148a, miRNA-200a, miRNA-200b, miRNA-200c, miRNA-216, miRNA-217, and miRNA-375 exhibited high expression levels in the normal pancreatic tissue compared with 33 other human tissues analysed in the same array. Alternatively, there were low expression levels with miRNA-133a, miRNA-143, miRNA-145, and miRNA-150 in cancerous tissue[60]. MiRNA-216 and miRNA-217 were found to have extensive infiltration in normal pancreatic tissues, and neglible levels in both cancerous tissue and pancreatic cell lines. The unique expression of these two miRNAs in normal pancreatic tissue samples compared to diseased tissues and cell lines renders a specificity for the pancreatic abundance. A global study also showed that both miRNA-216 and miRNA-217 were absent from 33 different human tissues [60]. MiRNA− 93, miRNA-196a, miRNA-196b, miRNA-203, miRNA-205, miRNA-210, miRNA− 221, miRNA-222 and miRNA-224 were upregulated only in cancerous tissues and cell lines. This observation is linked with a potential role in the neoplastic process. Special interest should be directed towards miRNA-196a and miRNA-196b as complete absence was observed in the normal and pancreatitis tissues. This gives a potential selectivity to pancreatic cancer as detailed in Table 1 [60].

**Table 1:** miRNAs as biomarkers in pancreatic cancer (“= high mainly in early lesions)

miRNA	Analysis Technique	Normal Pancreatic Tissues	Pancreatitis	PC	Cell lines	Reference
-20a, −29c 96, − 141,	Microarray	High	N/A	Low	N/A	[78]
-10a, −10b, − 204, − 372,	Microarray	Low	N/A	High	N/A	[82]
-93, −133a, − 203, − 205, − 210, − 224	Microarray	Low	N/A	High	High	[60]
-27a	Microarray	Low	N/A	High	N/A	[78]
-221, −222	Microarray, qRT-PCR	Low	N/A	High	High	[60, 79]
-216, −217	Microarray	Very high	N/A	Very low	Very low	[60, 78]
-200a	Microarray, TaqMan low density array, qRT-PCR	Low	N/A	High	N/A	[78, 81]
	Microarray	High		Low		[60]
-196a	Microarray, qRT-PCR	Absent	Absent	High	High	[60, 78, 79]
-15b, −95, −186, −190, −200b	qRT-PCR	Low	N/A	High	N/A	[79]
-146a	Microarray	Low	N/A	High	N/A	[78, 82]
−143, −145, −150	Microarray	Low	High	High	Absent	[60]
−141, −148a, −200b, −200c, −375	Microarray, TaqMan low density array, qRT-PCR	Low	N/A	N/A	High	[80]
−139, −142, −345	qRT-PCR	Low	N/A	Low	N/a	[76]
−100, −125b-1, −212, −301, −424	qRT-PCR	Low	N/A	High	N/a	[76]
−21	Microarray, TaqMan low density array, qRT-PCR	Low	N/A	High “	High	[77, 78, 80, 81]
−155	qRT-PCR	Low	N/A	High “	N/A	[77]
−205	qRT-PCR	Low	N/A	High “	N/A	[81]
−375	Microarray	High	Low	Low	Absent	[60]

### miRNAs as prognostic factors

Profiling miRNAs among patient samples with different disease characteristics and stages gives an understanding of the prognostic role of miRNAs, many of which are listed in Table 2. It was shown in a retrospective clinical study comprising 200 pancreatic ductal adenocarcinoma tissue samples (Department of Hepatobiliary and Pancreas Surgery of Seoul National University Hospital) that in a high-risk group (median survival time = 17.2 months) miRNA-574-5p, miRNA-1244, miRNA-4474-5p were upregulated. While miRNA-574-5p, miRNA-1244, miRNA-145-*, miRNA-328, miRNA-26b*, and miRNA-4321 showed association with overall survival (OS) and disease-free survival (DFS) [83]. Taubert and coworkers [84] have investigated the role of miRNA-155, miRNA-203, miRNA-210, miRNA-222, miRNA− 216, and miRNA-217 in overall survival. Upregulation of the first four of this group showed a correlation with poor prognosis and overall survival, while the same effect has been noticed with the downregulation of miRNA-217. In terms of miRNA-216, no effect has been detected in either up or downregulation. Tumour-related death increased 5.24-fold when the overexpression of miRNA-155, miRNA-203, miRNA-210, and miRNA-222 is combined [84]. miRNA-21 is also another indicator of poor outcome [85, 86]. Increased expression of miRNA-196a-2 resulted in a lower survival time (median survival time = 14.3 months) compared with downregulated expression (median survival time = 26.5 months) [86, 87]. Similar effects occurred with miRNA-219 as the median survival rate was 13.6 months in the overexpressing tumours, and 23.8 months in the tumours with downregulated miRNA-219 [88].

**Table 2:** miRNAs as prognostic factors and the associated targets and survival status in pancreatic cancer

miRNA	Expression in Pancreatic Cancer compared to normal tissue	Survival Status	Targets	Reference
-10b	Upregulated	Poor Survival	TIP30	[94, 95]
−21	Upregulated	Worse Survival	PTEN, PDCD4, IL-6R,CDK6	[85–87]
-34a	Upregulated	Better Survival	NOTCH, BCL2, CDK6	[96–98]
−155	Upregulated	Poor Survival	TP53INP	[99, 100]
-let-7 Family	Downregulated	Poor Survival	KRAS, HRAS, TRIM71	[101, 102]
−200 Family	Downregulated	Better Survival	E-cadherin, ZEB	[91, 96, 103]
−216	Downregulated	Poor Survival	ROCK1	[104, 105]

Furthermore, miRNAs could play a prolific role in the prediction of chemoresistance and responsiveness to different therapeutic approaches. For example, several markers indicate the degree of responsiveness, where miRNA-320c is a prognostic factor for gemcitabine clinical response prediction [89]. Moreover, post-surgery survival rates were higher in patients with increased levels of miRNA-200c (MST = 42 months, 5-year survival rate = 33.5%) than the lower miRNA-200c expressing individuals (MST = 19 months, 5-year survival rate = 11.2%). This observation correlated with low invasiveness of cells after the upregulation of miRNA-200c following in vitro experiments [90].

Irregular expression of miRNA was found in the development of a gemcitabine-resistant cell line. miRNA-200b, miRNA-200c, let-7b, let-7c, let-7d and let-7e were downregulated in gemcitabine-resistant cells [91]. Similarly, miRNA-33a is also downregulated in gemcitabine-resistant cells, and upon the restoration of normal levels, gemcitabine sensitivity was restored in the MIA-PaCa2 PC cell line [92]. Increased levels of miRNA-320c in gemcitabine-resistant cells suggests incorporation through regulation of SWI/SNF Related Matrix Associated Actin Dependent Regulator Of Chromatin Subfamily C Member 1 (SMARCC1), as benefits from the gemcitabine regimen were only found among the patients expressing SMARCC1. miRNA-205 was used in combination with gemcitabine as a chemosensitiser through decreased production of caveolin-1 and Ki-67 [93]. Orthotopic pancreatic mice were implanted with either lenti-hsamiRNA205 treated MIA PaCa-2R cells, or lenti-hsamiRNAScramble treated MIA PaCa-2R cells. Following intravenous delivery of GEM-conjugated polymeric micelles thrice weekly for two weeks at a concentration of 40 mg/kg, it was observed that in the lenti-hsamiRNA205 treated cells there was a significant reduction in tumour growth. These mice bore tumours with average volumes of 77.83 ± 21 mm, compared to mice implanted with lenti-hsamiRNAScramble treated cells, which exhibited tumours of volumes of 172.85 ± 17 mm.

## Conclusions

Due to the aggressive nature of PC and the lack of biomarkers, miRNAs represent a promising tool to help in the development of prediction, managing, and treating agents to improve low survival rates. The therapeutic potential of miRNAs can be implicated after investigating downstream regulatory mechanisms observed among different molecular pathways, as some can play a tumour suppressive role and others are oncomiRNAs. The restoration of these miRNAs levels to that of healthy tissue could therefore be beneficial in maintaining the endogenous anti-tumour regulatory mechanisms.

However, large-scale clinical studies need to be explored to establish clinical relevance of the collected data. To date, one clinical trial is ongoing studying miRNA-25 as a diagnostic tool in PC (NCT03432624) [106]. Variations in results among studies arise from using pancreatic specimens, which contain heterogenous cell populations. These cell types include the ductal, acinar, and islet cells, along with other inflammatory, fibroblastic components that will accompany the tumour development. Prediction of target genes of different miRNAs could be considered the major drawback of assigning miRNA in large scale applications, as the predictive algorithms give an enormous number of targets for a single miRNA. Attention should be given to the miRNA’s mechanism of action, as more innovative methods will be required to validate the predicted targets.

RNAs are gaining momentum as therapy options. MRX34, for example, is a miRNA-34 mimic encapsulated within a lipid-based vessel known as NOV40. MRX34 was utilised in a multicentre phase I clinical trial (2013) for the treatment of patients with primary liver cancer, melanoma, lymphoma, small cell lung cancer, multiple myeloma or renal cell carcinoma. By June 2016, 99 patients were recruited onto the trial with HCC, NSCLC or pancreatic cancers. Following completion of the trial, 3 patients achieved prolonged confirmed partial responses. Moreover, 14 patients presented with stable disease (median duration- 136 days) [107]. An example of an FDA approved RNAi based therapy by Alnylam Pharmaceuticals is Onpattro™ for the treatment of polyneuropathy of hereditary transthyretin-mediated amyloidosis in adults. Onpattro™ contains patisaran, which comprises a siRNA conjugated with a lipid complex. The drug’s mechanism of action results in binding with the TTR protein. The reduction in TTR protein levels in the liver results in a drop in amyloid deposits. The FDA approval of Onpattro™ in August 2018 was the result of a successful Phase III clinical trial dubbed APOLLO. The trial had 225 patients enrolled, 148 of which received Onpattro™ once every three weeks (0.3 mg/ kg body weight), while the remaining patients received a placebo drug. Patients receiving Onpattro™ displayed improvements, with 51% of patients exhibiting an improved quality of life (measured using the Norfolk Quality of Life Diabetic Neuropathy (QoL-DN)), as opposed to only 10% in the placebo control [108].

MiRNAs hold potential as innovative gene therapies but success is heavily reliant on an efficient delivery vector [109, 110]. If indeed miRNAs are to be used in pancreatic cancer, the delivery systems must transport the cargo to the destination site, not evoke an immune response and not have prohibitive production costs so that wide-spread adoption of the these nanotherapies can be realised.

## Data Availability

Not Applicable.

## Abbreviations

BRCA2: Breast Cancer A2
CDKN2A: Cyclin-dependent Kinase
DM: Diabetes Mellitus
DNA: Deoxyribonucleic acid
eIF4E: Eukaryotic Initiation Factor 4E
ERK: Extracellular Signal-regulated Kinase
FNA: Fine Needle Aspiration
GAP: GTPase Activating Protein
GTP: Guanosine Triphosphate
K-RAS: Kirsten Rat Sarcoma
LDH: Lactate Dehydrogenase
MAPK: Mitogen-activated Protein Kinase
miRNA: micro Ribonucleic acid
mRNA: messenger Ribonucleic acid
NE: Norepinephrine
oncomiR: Ongogenic micro Ribonucleic acid
PanIN: Pancreatic Intraepithelial Neoplasia
PC: Pancreatic Cancer
PDGFRb: Platelet-derived Growth Factor Receptor b
PI3K: Phosphoinositide-3-kinase
RISC: RNA-induced silencing complex
RNA: Ribonucleic acid
SMAD: Mothers Against Decapentaplegic
SMARCC1: Chromatin Subfamily C Member 1
TGFβ: Transforming Growth Factor β
TβRII: Threonic Kinase Receptor II
UTR: Untranslated Region
